# Effect of Body-Weight-Based Resistance Training on Balance Ability and Fear of Falling in Community-Dwelling Older Japanese Women

**DOI:** 10.3390/sports13010008

**Published:** 2025-01-07

**Authors:** Zhenyue Liu, Shuji Sawada, Pengyu Deng, Hisashi Naito, Shuichi Machida

**Affiliations:** 1 Graduate School of Health and Sports Science, Juntendo University, Chiba 270-1695, Japan; sh4222011@juntendo.ac.jp (Z.L.); sh-sawada@juntendo.ac.jp (S.S.); houu.tou@gmail.com (P.D.); hnaitou@juntendo.ac.jp (H.N.); 2 Institute of Health and Sports Science & Medicine, Juntendo University, Chiba 270-1695, Japan; 3 Faculty of Health and Sports Science, Juntendo University, Chiba 270-1695, Japan

**Keywords:** low-load resistance training, fall prevention, physical function, static balance, concern about falling

## Abstract

Background: This study aimed to investigate the effects of a 12-week body-weight-based resistance training program on balance ability and fear of falling in community-dwelling older women. Methods: Twenty-three older women were assigned to either an intervention group that performed the low-load resistance training with slow movement using the body weight (LRT group; *n* = 12) or a control group (CON group; *n* = 11). The LRT group participated in the exercise session twice weekly for 12 weeks, while the CON group maintained their daily routine. The 30 s chair stand test (CS-30) was applied to measure lower-extremity muscle strength, balance ability was evaluated using one-leg standing tests with eyes open (OLST-O) and closed (OLST-C), and fear of falling among all participants was assessed using the Falls Efficacy Scale International (FES-I) before (pre) and after (post) the intervention. A two-way analysis of variance with repeated measures [group (LRT and CON) × time (pre and post)] was carried out to evaluate the intervention effects. Results: Significant interactions were observed in the CS-30 (F = 9.503, *p* < 0.01, $\eta_{p}^{2}$ = 0.312), OLST-O (F = 5.211, *p* < 0.05, $\eta_{p}^{2}$ = 0.199), and OLST-C (F = 5.257, *p* < 0.05, $\eta_{p}^{2}$ = 0.200), though significant simple main effects from pre to post were observed only in the LRT group. The CS-30 scores (pre: 19.8 ± 3.8 times, post: 25.5 ± 5.6 times; *p* < 0.001), OLST-O time (pre: 78.8 ± 35.8 s, post: 96.2 ± 29.9 s; *p* < 0.01), and OLST-C time (pre: 10.2 ± 5.9 s, post: 17.4 ± 12.2 s; *p* < 0.01) were improved before and after the intervention. However, a significant interaction was not observed in FES-I (F = 1.335, *p* = 0.261, $\eta_{p}^{2}$ = 0.06). Conclusions: The 12-week body-weight-based resistance training program enhanced lower-extremity muscle strength and balance ability but did not lessen the fear of falling in community-dwelling older women. The study findings offer relevant information for fall prevention in older adults.

## 1. Introduction

As the global population ages, extending the health span (i.e., healthy life expectancy) of older adults has become one of the most critical issues [1]. Falls are associated with morbidity and high mortality, meaning they are considered a significant public health problem that hinders the attainment of a long health span among older adults [2]. Furthermore, for women, muscle strength and physical function decline severely with age, and the risk of falling is higher compared to in men [3]. Therefore, fall prevention is essential for older women and appropriate strategies need to be developed.

Numerous multidimensional factors contribute to falls among older adults [4,5]. Lower-extremity muscle strength [6] and balance ability [7], as objective physical factors, are strongly associated with the risk of falling and have been the primary focuses of research. In addition, fear of (concern about) falling (FOF), a subjective psychological factor associated with falls, is considered to be equally crucial to falls [8]. This concern is prevalent among older adults irrespective of whether they have previously experienced a fall [8,9]. Lower-extremity muscle weakness and balance ability decline correlate with the severity of FOF in community-dwelling older adults [10,11]. A FOF may restrict physical activity [12], accelerating physical functional decline, such as muscle weakness and balance issues [13], increasing the risk of sarcopenia and frailty [14,15], and subsequently exacerbating the FOF [16], resulting in a vicious cycle. Hence, from the perspective of fall prevention and healthy longevity in older adults, preventing and improving the deterioration of lower-extremity muscle strength and balance ability as objective physical functions, as well as addressing the FOF as a subjective psychological factor, are essential [17].

Exercise training has been proposed as an effective intervention strategy to improve muscle function [18] and balance ability [19] and reduce the FOF [20] in older adults, with various effective exercise types reported. Resistance training is particularly effective; although it is generally used to improve muscle function, it has also been reported to improve balance [21,22,23,24] and reduce the FOF [25] in older people. However, most resistance training programs require a high load, which may not generalize well among older people. Hence, a better practical resistance training program is required. Recently, the effectiveness of low-load resistance training with slow movement using the body weight (LRT) in increasing muscle mass and strength has been reported [26,27,28,29]. However, the beneficial effects of LRT on balance ability and the FOF have not been thoroughly investigated. This is a pressing research gap to fill, as LRT can be implemented conveniently and safely [27], meaning it could be applied to older people living in the community.

Therefore, this study aimed to investigate the effects of a 12-week LRT intervention on lower-extremity muscle strength and balance ability as the objective physical factors, and the FOF as a subjective psychological factor, in healthy older women. We hypothesized that 12 weeks of LRT would simultaneously enhance lower-extremity muscle strength and balance ability, as well as lessen the FOF.

## 2. Materials and Methods

### 2.1. Participants and Study Design

**Figure 1:**
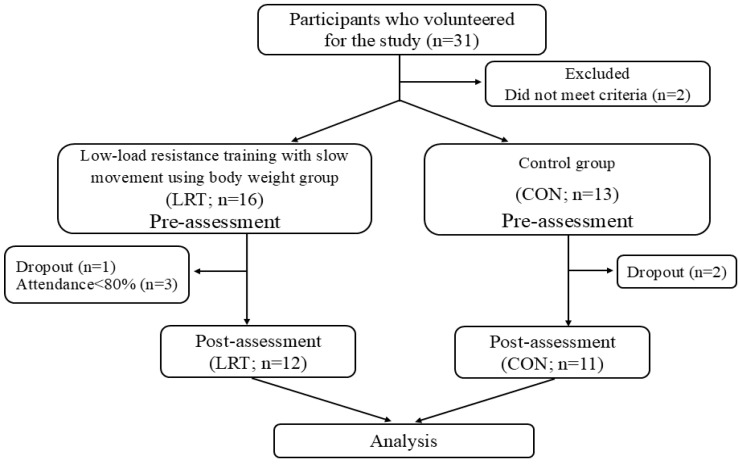
Flow of participants through the trial.

Thirty-one community-dwelling older Japanese women were recruited from the local community through printed advertisements. The inclusion criteria were Japanese women living independently in the community, who were able to complete the measurements and exercise intervention, as judged by the physician-in-charge. All recruits were informed of this study’s methods, procedures, and risks and provided written informed consent before participating. The exclusion criteria for this study were severe cardiovascular or cerebrovascular diseases, activity limitations due to bone or joint diseases within the past six months, lack of approval from their regular physicians, and inability to follow our instructions. Individuals who met these criteria were excluded, and twenty-nine participants qualified for this study. The participants were assigned to either an intervention group that performed low-load resistance training with slow movement using the body weight (LRT group; *n* = 16) or a control group (CON group; *n* = 13). Assessments were conducted to evaluate all participants’ body characteristics and physical/psychosocial-related parameters before (pre) and after (post) the 12-week intervention period. The LRT group participated in the exercise session during the intervention period, while the CON group maintained their daily routine without participating in the exercise. All participants were instructed to avoid engaging in other physical activities throughout the trial. Through the 12-week intervention, one participant in the LRT group dropped out of the exercise sessions for personal reasons, and two in the CON group were absent from the post-assessment. In addition, three participants in the LRT group were excluded from statistical analyses for having less than 80% attendance of the exercise sessions. Therefore, 12 participants in the LRT group (mean age: 67.3 ± 4.8 years) and 11 in the CON group (mean age: 69.4 ± 6.0 years) were included in the final analysis (Figure 1). The required sample size was calculated using G*power software (version 3.1.9.6, Düsseldorf, Germany) (ANOVA: repeated measures, within–between interaction) and with a medium effect size (partial η^2^ of 0.09) [30], α err prob of 0.05, and statistical power (1 − β err prob) of 0.80. The calculation indicated that 22 participants were needed (11 in each group). This study was conducted under the Declaration of Helsinki and was approved by the Ethics Committee for Human Experiments of Juntendo University (approval number: 2022-73). Experiments were conducted and data were collected from August 2022 to December 2022.

### 2.2. Training Program

The low-load resistance training program, mainly using body weight, was conducted twice a week for 12 weeks (a total of 22 sessions) through group exercise sessions at least 48 h apart. The program comprised nine resistance training exercises: squat, split squat, push-up, heel raise, crunch, hip lift, seated row, shoulder press, and arm curl. The first six exercises used the participant’s body weight, and the last three used elastic bands (Thera-Band^®^; The Hygenic Corporation, Akron, OH, USA). In the first two weeks (first four sessions) of the intervention period, the participants only performed four exercises: squat, push-up, crunch, and hip-lift, with each trained for three sets of eight repetitions, with a 60 s rest between each set. They were instructed to perform each concentric and eccentric phase of movements slowly (spending 3 s on each phase). From the third week onward, the number of exercises per session, sets per exercise, repetitions per set, and program time gradually increased every two weeks, and the rest interval gradually decreased every two weeks. The final program with all nine training exercises involved three sets of 15 repetitions each with 30 s of rest between sets, taking roughly 90 min per session (including warm-up and cool-down stretches). In each exercise class, three trained instructors were involved in the instruction, working to confirm the participants’ movements and enable them to perform the program correctly. Participants were instructed to record their fitness condition and rating of perceived exertion during each training session. The training program was conducted according to the protocol of a previous study [27].

### 2.3. Measurements

#### 2.3.1. Lower-Extremity Muscle Strength

The 30 s chair stand test (CS-30) assessed the lower-extremity muscle function. Participants were instructed to complete sit-to-stand movements without using their arms as many times as possible from a 40 cm high seat in 30 s, while trained examiners controlled the timer and recorded the number of times participants completed the task. The CS-30 test can be used to evaluate lower-extremity muscle strength in older Japanese adults [31], and our previous study reported that the test–retest reliability using the ICC was 0.78 [27].

#### 2.3.2. Balance Ability

The one-leg standing tests assessed balance function with both eyes open (OLST-O) and eyes closed (OLST-C). Participants performed both the OLST-O and OLST-C tests barefoot on a flat, hard floor after adequate practice. They chose which leg to stand on according to their preference. Throughout the tests, two trained examiners stood nearby, one acted as timekeeper and the other on hand to prevent any falls or injuries due to loss of balance. Participants were instructed to stand facing a wall more than one meter away and balance on the chosen leg without assistance, keeping their hands on their hips. The test was terminated under the following conditions: if the lifted leg touched the supporting leg or the floor, the supporting leg moved out of position, or either hand left the hip [32,33]. To avoid the ceiling effect as much as possible, the maximum measurement time was set to 120 s for each test, and participants were given two trials unless they could complete 120 s on the first in both tests. The better of the two trial times in both tests were used for the analysis. The same leg was used in the post-assessment and pre-assessment to ensure the testing condition. The previous study reported that the test–retest reliabilities when using the ICC were 0.90 for the OLST-O and 0.74 for the OLST-C [34], and the ICC of OLST-C was found to be 0.83 in our study.

#### 2.3.3. Fear of (Concerns About) Falling (FOF)

FOF was assessed with the Falls Efficacy Scale International (FES-I), which consists of 16 items regarding the level of concern about falling when carrying out indoors and outdoors activities of daily living and social participation. Each item was scored according to a 4-point Likert scale (1 = not at all concerned, 4 = very concerned) [35]. We used the Japanese version of FES-I; the reported ICC was 0.79–0.87 [36,37].

### 2.4. Statistical Analysis

The variables were presented as means and standard deviations (SD). Comparisons of participants’ physical characteristics and outcomes between the LRT and CON groups at baseline (pre-assessment) were conducted using Mann–Whitney U tests and unpaired t-tests. To compare pre–post changes in the LRT and CON groups, two-way analysis of variance (ANOVA) with repeated measures [group (LRT and CON) × time (pre and post)] was carried out, and simple main effects analysis (Bonferroni correction for the post hoc test) was performed when a significant interaction between the group and time was found in the two-way ANOVA. The effect size of the two-way ANOVA was assessed using the partial η^2^ ($\eta_{p}^{2}$), with a value of 0.09 interpreted as a medium effect size [30]. In addition, to exclude the confounding effect of baseline differences in the intervention effect analysis, an analysis of covariance (ANCOVA) was performed. Statistical analyses were performed using SPSS software (version 29.0.0; IBM Japan, Tokyo, Japan). Statistical significance was set at *p* < 0.05.

## 3. Results

Table 1 shows the baseline characteristics of the participants. Significant baseline differences in physical characteristics and outcomes between groups were not observed in the pre-assessment (Table 1).

Table 2 shows the changes and the comparisons of CS-30, OLST-O, OLST-C, and FES-I before and after the 12-week intervention in both groups. Significant group × time interactions were observed in CS-30 (F = 9.503, *p* < 0.01, $\eta_{p}^{2}$ = 0.312), OLST-O (F = 5.211, *p* < 0.05, $\eta_{p}^{2}$ = 0.199), and OLST-C (F = 5.257, *p* < 0.05, $\eta_{p}^{2}$ = 0.200) but not in FES-I (F = 1.335, *p* = 0.261, $\eta_{p}^{2}$ = 0.06). Regarding the results of a simple main effects analysis, significant simple main effects from pre to post were observed only in the LRT group, and not in the CON group, while the CS-30 time (pre: 19.8 ± 3.8 times, post: 25.5 ± 5.6 times; *p* < 0.001), OLST-O time (pre: 78.8 ± 35.8 s, post: 96.2 ± 29.9 s; *p* < 0.01), and OLST-C time (pre: 10.2 ± 5.9 s, post: 17.4 ± 12.2 s; *p* < 0.01) were improved before and after the intervention.

**Figure 2:**
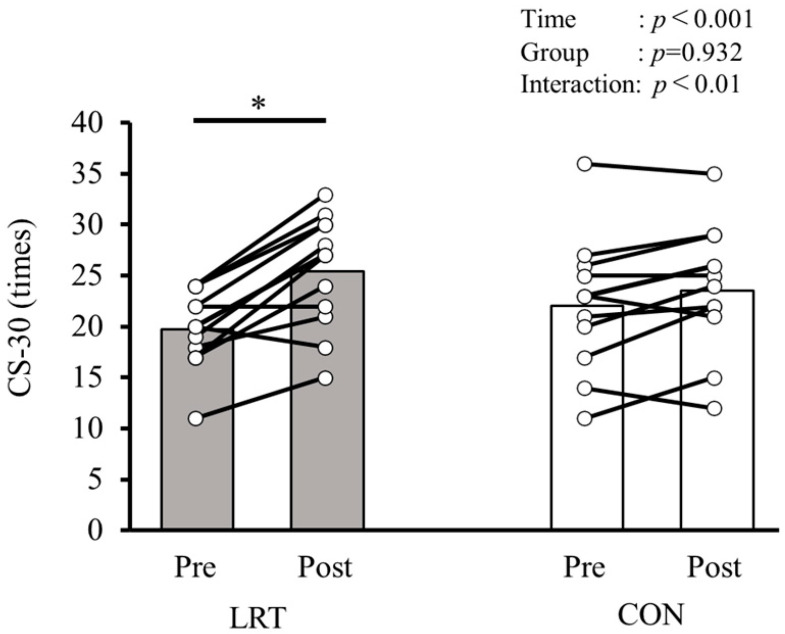
Comparison of CS-30 before and after the 12-week intervention in both groups. LRT, group that performed low-load resistance training with slow movement using the body weight; CON, control group; Pre, before intervention; Post, after intervention; CS-30, 30 s chair stand test. * Significantly greater than Pre (*p* < 0.001).

Figure 2 shows a comparison of CS-30 before and after the 12-week intervention in both groups. A significant group × time interaction (F = 9.503, *p* < 0.01, $\eta_{p}^{2}$ = 0.312) and main effect of time (F = 29.104, *p* < 0.001, $\eta_{p}^{2}$ = 0.581) were observed, while a main effect of the group (F = 0.007, *p* = 0.932, $\eta_{p}^{2}$ < 0.001) was not observed. When comparing the intervention effect of the LRT and CON groups using simple main effects analyses, a significant increase from pre to post in LRT was observed (pre: 19.8 ± 3.8 times, post: 25.5 ± 5.6 times; *p* < 0.001), but not in the CON group (pre: 22.1 ± 6.8 times, post: 23.6 ± 6.5 times; *p* = 0.124; Table 2).

**Figure 3:**
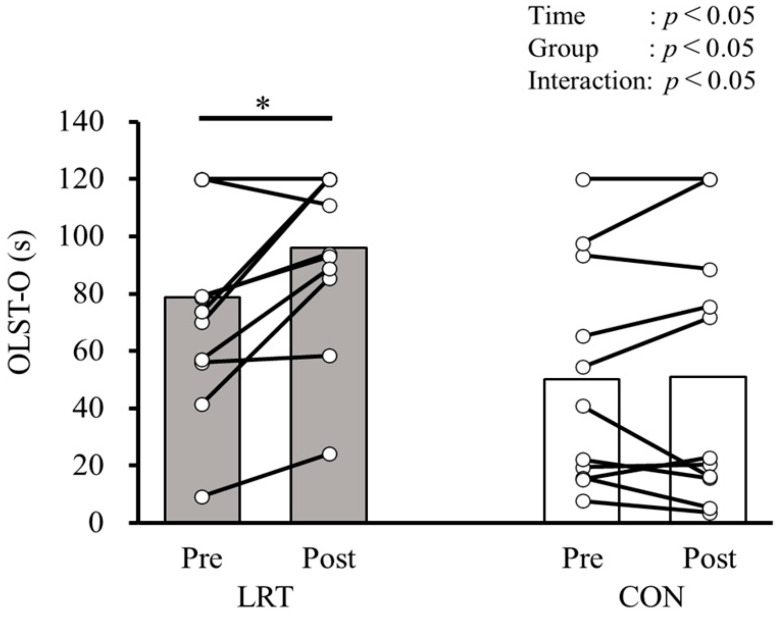
Comparison of OLST-O before and after the 12-week intervention in both groups. LRT, group that performed low-load resistance training with slow movement using the body weight; CON, control group; Pre, before intervention; Post, after intervention; OLST-O, one-leg standing balance test with eyes open. *, significantly greater than Pre (*p* < 0.01).

Figure 3 shows a comparison of OLST-O before and after the 12-week intervention in both groups. A significant group × time interaction was observed (F = 5.211, *p* < 0.05, $\eta_{p}^{2}$ = 0.199), and significant main effects of time (F = 6.186, *p* < 0.05, $\eta_{p}^{2}$ = 0.228) and the group (F = 5.827, *p* < 0.05, $\eta_{p}^{2}$ = 0.217) were also observed. When detecting the intervention effect in OLST-O using simple main effects analyses, the performance of OLST-O was only significantly improved in the LRT group (pre: 78.8 ± 35.8 s, post: 96.2 ± 29.9 s; *p* < 0.01), and not in the CON group (pre: 50.2 ± 39.1 s, post: 50.9 ± 45.2 s; *p* = 0.889; Table 2).

**Figure 4:**
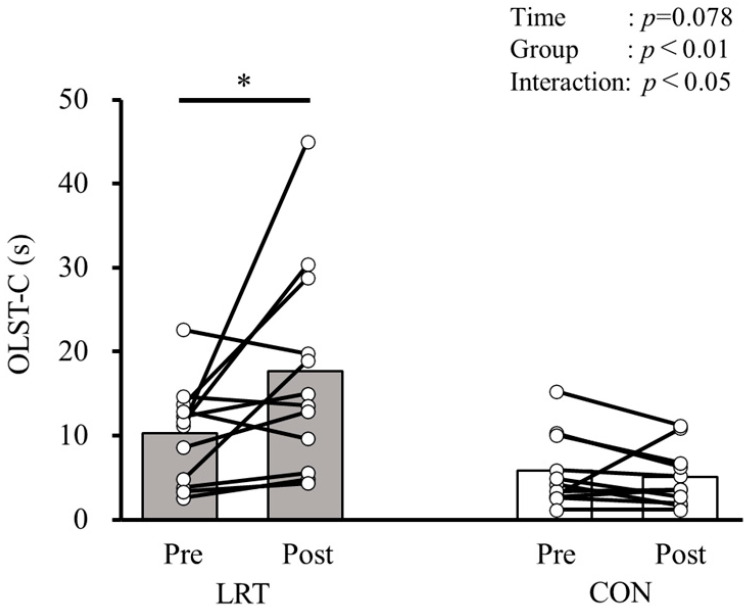
Comparison of OLST-C before and after the 12-week intervention in both groups. LRT, group that performed low-load resistance training with slow movement using the body weight; CON, control group; Pre, before intervention; Post, after intervention; OLST-C, one-leg standing balance test with eyes closed. *, significantly greater than Pre (*p* < 0.01).

Figure 4 shows a comparison of OLST-C before and after the 12-week intervention in both groups. A significant group × time interaction (F = 5.257, *p* < 0.05, $\eta_{p}^{2}$ = 0.200) and group main effect (F = 10.676, *p* < 0.01, $\eta_{p}^{2}$ = 0.337) were observed, but not a time main effect (F = 3.440, *p* = 0.078, $\eta_{p}^{2}$ = 0.141). When detecting the intervention effect in OLST-C using simple main effects analyses, the performance of OLST-C was also only significantly improved in the LRT group (pre: 10.2 ± 5.9 s, post: 17.4 ± 12.2 s; *p* < 0.01), and not in the CON group (pre: 5.8 ± 4.3, post: 5.0 ± 3.5; *p* = 0.765; Table 2).

**Figure 5:**
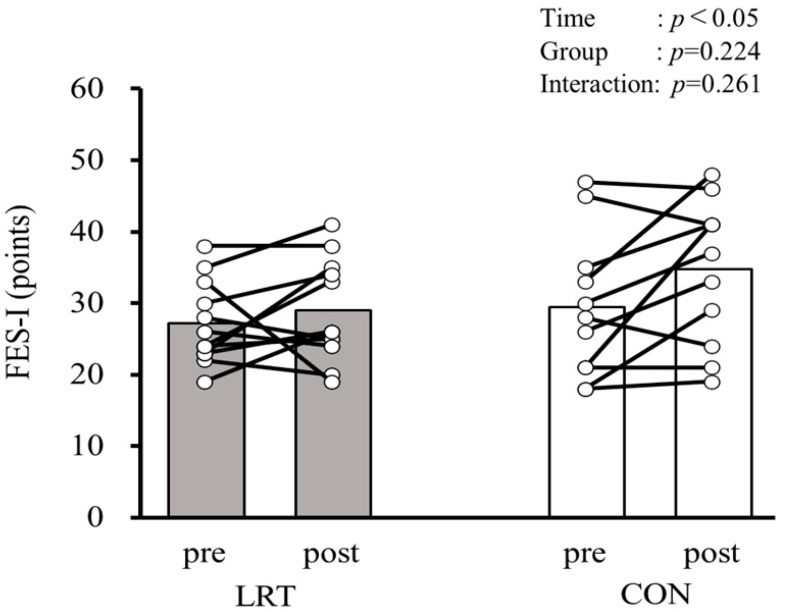
Comparison of FES-I scores before and after the 12-week intervention in both groups. LRT, group that performed low-load resistance training with slow movement using the body weight; CON, control group; Pre, before intervention; Post, after intervention; FES-I, Falls Efficacy Scale International.

The results of FES-I are shown in Figure 5, which reveals that a significant group × time interaction (F = 1.335, *p* = 0.261, $\eta_{p}^{2}$ = 0.06) was not observed. A significant main effect was observed for time (F = 5.305, *p* < 0.05, $\eta_{p}^{2}$ = 0.202) but not for the group (F = 1.572, *p* = 0.224, $\eta_{p}^{2}$ = 0.07).

To exclude the influence of baseline differences as a confounding factor in the intervention effect analysis, an analysis of covariance (ANCOVA) was performed for each outcome with the baseline value of each outcome as a covariate. Significant group differences in post-intervention scores were observed in CS-30 (F = 8.132, *p* = 0.010), OLST-O (F = 5.981, *p* = 0.024), and OLST-C (F = 5.491, *p* = 0.030) but not in FES-I (F = 2.122, *p* = 0.161).

## 4. Discussion

This study investigated the effects of a 12-week LRT intervention on lower-extremity muscle strength and balance ability as objective physical factors and the FOF as a subjective psychological factor. Two-way ANOVA and ANCOVA were used to assess the intervention effects, with intervention effects of CS-30, OLST-O, and OLST-C observed using both statistical methods, while one for FES-I was not. The results showed that the 12-week intervention enhanced the physical aspects, lower-limb muscle strength and balance ability, as evaluated by CS-30 and OLST, while no significant improvement in the psychological aspect, the FOF, was observed.

CS-30 was used to evaluate the lower-limb muscle strength of the participants in this study, and a significant interaction between the two groups was observed, which suggested that a 12-week, twice-weekly exercise intervention using LRT improved lower-limb muscle strength in older women. This corresponds with the results of our previous study, which used the same exercise program for 12 weeks of exercise; after the exercise intervention, we observed an increase in CS-30, lower-limb muscle strength, and muscle thickness [27]. Although muscle morphology measurements such as muscle thicknesses using ultrasound or DXA and lower-extremity muscle strength evaluated with an ergometer were not included in the present study, given the confirmation we found of the correlation of CS-30 with lower-extremity muscle strength [31] as well as with thigh muscle thickness [38], we consider there to be a high likelihood that improvement was produced in the muscle function (muscle mass and muscle strength) of the participants of this study. In addition, different from our previous intervention study [27], we established a control group that did not receive the exercise intervention, and we observed significant changes only in the intervention group and not in the control group after the intervention, which indicated the effectiveness of the LRT intervention in enhancing lower-extremity muscle strength. Furthermore, the present study focused on the effects on balance, which extends the evidence from our previous research [27].

In this study, we observed that the performance of OLST-O and OLST-C, which are the balance factors in objective physical fitness, was enhanced through the 12-week intervention. The OLST is widely used in clinical or community settings as the primary indicator of static balance and postural control [32,39], and it can predict falls [40] and future survival [41]. Several studies have shown that resistance training effectively enhances performance (prolonged standing time) in OLST-O. Marques et al. [22] showed that full-body training (quadriceps, hamstrings, gluteal, trunk, abdominal, and arms) with machines three times per week for 32 weeks, with an intensity of 50–80% 1RM, could improve OLST-O performance in older adults. Gonzalez et al. [21] used body weight and machines to perform progressive full-body resistance training, which also enhanced OLST-O performance in older adults, although it was carried out only at a frequency of twice a week for six weeks. In our study, LRT with a progressively increasing intensity was performed twice a week for 12 weeks, with the primary load being the participant’s own body weight. No special training equipment was used; as LRT has a relatively long duration of muscle contraction as the slow movement, it may potentially affect static balance even at a low intensity. In another previous study with active older adults, an LRT intervention (3 s concentric, 3 s eccentric, and 1 s isometric actions; no rest between each repetition) was performed once a week for 16 weeks, but no enhancement of muscle function (muscle strength and muscle size) or balance (OLST-O) was observed [29], which is in contrast to our results. This is probably due to the previous study conducting LRT with only five exercises, each performed as a single set once a week, despite participants having a high static balance level (OLST-O: 81.0 ± 40.0 s) before the intervention [29]. In contrast, this study performed LRT with nine exercises in three sets twice a week. Thus, the higher intensity, frequency, and duration of muscle contraction may have contributed to the effect on static balance. Furthermore, OLST-O performance (time) can be used as a predictor of low muscle mass [42]; another previous study showed that muscle thickness in the core and lower limbs is a determinant of static balance [43]. Although muscle morphometry was not performed in the present study, we observed increased abdominal and anterior thigh muscle thicknesses in our previous study using the same exercise program [27]. In the present study, several exercises targeted the quadriceps and abdominal muscles, such as squats, split squats, and crunches; in particular, squats and crunches were performed throughout the intervention period.

Regarding OLST-C, to the best of our knowledge, this is the first study to investigate the effect of LRT on OLST-C, and we observed a significant increase in OLST-C time after 12 weeks of the intervention. Katsura et al. [44] showed that 4–6 basic manual resistance exercises focusing on eccentric muscle contractions (more load for the eccentric phase) three times per week for eight weeks improved the balance with the eyes closed, and calf muscle training, especially with eccentric contractions (slow drop of the heel), may improve proprioception and probably contributes to the balance with the eyes closed. The exercise program in our study also included calf muscle training (heel raise), and the slow descent and ascent of the movement (3 s eccentric and 3 s concentric contractions), which included longer eccentric contractions, was emphasized. In addition, our program also included the squat and split squat as an ascending–descending movement. These slow contractions may impact balance and proprioception, especially in squats, split squats, and heel raises, as these movements require postural control to prevent balance disruption. Future research is needed to investigate the effects of LRT on proprioception and other balance indicators (i.e., parameters related to the center of pressure), to clarify the mechanism of its effects on balance.

In this study, the FES-I scale was used to evaluate the FOF (or concern about falling), and no improvement in the FOF was found after the 12-week LRT intervention. In a previous study, FES-I scores above 27 points were defined as a high degree of FOF [9], and we considered that the participants in this study may have a high FOF at the baseline, although they had a certain degree of physical function. The world guidelines for falls prevention report that the FOF is an important risk factor for falls and recommend the use of a standardized instrument, FES-I or short FES-I, to assess the FOF or concern about falling in community-dwelling older adults (Grade 1A) [45]. Regarding FOF interventions, recently, two systematic reviews demonstrated the effectiveness of exercise interventions for reducing the FOF, with large effect sizes and focuses on holistic exercise (i.e., Taichi, Pilates, or yoga) [46] and balance training [20]. However, the optimal prescription of FOF improvement exercises for older adults is unclear [20]. To our knowledge, this study is the first to investigate the effect of LRT on the FOF, and an improvement was not observed after 12 weeks of LRT intervention. Several studies have reported the effects of resistance training on the FOF. Yamada et al. [25] used 10-RM for three sets of 10 repetitions for each machine form of resistance training (seated row, leg press, leg curl, and leg extension) twice a week for 50 weeks in frail and robust older adults. The FOF assessed by FES improved after the duration of the resistance training intervention only in frail older adults. Pirauá et al. [47] investigated the effect of 24 weeks of three-times-per-week resistance training (2–5 sets and 7–12 repetitions; leg press, horizontal dumbbell chest press, unilateral row with dumbbells, plank, bridge, and abdominal exercises) on the FOF measured using FES-I in healthy older adults, but no improvement in the FOF was observed at 12 and 24 weeks of the intervention. The discrepancy in results of the previous studies and our study may be mostly explained by the duration of the intervention (50 weeks vs. 24 or 12 weeks), as contact time with the intervention instructor was reported to be effective in reducing the FOF in a previous review [46]. Furthermore, the discrepancy in effects of the resistance training may be due to differences in participants and the intensity of the exercise. Our study included only female participants, and women are generally reported to have a higher FOF [16]. The effect of exercise interventions on the FOF in different sexes remains uncertain. Future studies should address this issue, and a systematic review is needed to summarize the effects of resistance exercise on the FOF.

In addition, exercise interventions may focus more on enhancing and improving the objective physical function. To lessen the FOF, which is a subjective psychological factor, cognitive behavioral therapy (CBT) is also an effective approach. The core components of this approach include cognitive restructuring, personal goal setting, and promotion of physical activity [48]. In our study, we observed an improvement in objective physical function, such as lower-limb muscle strength and balance, but not in the FOF. This may be due to the short duration of the intervention and participants’ unawareness of the changes in their objective abilities, thus maintaining their concern about falling. CBT is expected to be effective as an adjunctive therapy to exercise interventions [48], as cognitive restructuring may be effective in helping older adults to correctly understand their physical abilities, reducing their concern about falling during daily activities, and better promoting physical activity (i.e., maintain LRT after the intervention), forming a virtuous cycle. Therefore, future studies should investigate the combined effect of LRT and CBT in lowering the FOF, while improving physical aspects such as leg strength and balance [49], thus better achieving the goal of fall prevention.

Several limitations of this study should be noted. First, the participants of this study included only healthy women who were community-dwelling, and the sample size was small. Thus, the applicability of the results of this study to other populations is uncertain. Second, this study assessed lower-extremity muscle strength and balance based on associated physical function. Precision instruments such as isometric ergometers and force platforms were not used, and only static balance was assessed. However, it should be noted that the convenience of the CS-30 and OLST make them highly appropriate for community assessment or self-assessment by older adults. Thirdly, although we set up a control group, it was not randomly assigned; a randomized controlled trial design is needed in future studies to better validate the intervention.

## 5. Conclusions

Our results indicate that the 12-week body-weight-based resistance training program improved the physical aspects of lower-limb muscle strength and balance in community-dwelling older women but did not improve the FOF. The study findings provide relevant information on fall prevention in older adults. Future studies should investigate the effect of a multicomponent intervention on the fear of falling.

## Figures and Tables

**Table 1:** Characteristics of participants.

	LRT (*n* = 12)	CON (*n* = 11)	*p*-Value
Age (years)	67.3 ± 4.8	69.4 ± 6.0	0.109
Height (cm)	153.4 ± 4.9	157.4 ± 6.0	0.099
Weight (kg)	54.0 ± 6.6	57.4 ± 9.1	0.321
BMI (kg/m^2^)	23.0 ± 2.7	23.3 ± 4.0	0.829
CS-30 (times)	19.8 ± 3.8	22.1 ± 6.8	0.347
OLST-O (s)	78.8 ± 35.8	50.2 ± 39.1	0.082
OLST-C (s)	10.2 ± 5.9	5.8 ± 4.3	0.069
FES-I (points)	27.1 ± 5.8	29.3 ± 10.1	0.537

Data are shown as mean ± SD. LRT, group that performed low-load resistance training with slow movement using the body weight; Con, control group; BMI, body mass index; CS-30, 30 s chair stand test; OLST-O, one-leg standing balance test with eyes open; OLST-C, one-leg standing balance test with eyes closed; FES-I, Falls Efficacy Scale International.

**Table 2:** Comparison of CS-30, OLST-O, OLST-C, and FES-I before and after the 12-week intervention in both groups.

	LRT (*n* = 12)		CON (*n* = 11)		*p*-Value		
	Pre	Post	Pre	Post	Time	Group	Interaction
CS-30 (times)	19.8 ± 3.8	25.5 ± 5.6 **	22.1 ± 6.8	23.6 ± 6.5	<0.001	0.932	<0.01
OLST-O (s)	78.8 ± 35.8	96.2 ± 29.9 *	50.2 ± 39.1	50.9 ± 45.2	<0.05	<0.05	<0.05
OLST-C (s)	10.2 ± 5.9	17.4 ± 12.2 *	5.8 ± 4.3	5.0 ± 3.5	0.078	<0.01	<0.05
FES-I (points)	27.1 ± 5.8	28.7 ± 7.1	29.3 ± 10.1	34.5 ± 10.1	<0.05	0.224	0.261

Data are shown as mean ± SD. LRT, group that performed low-load resistance training with slow movement using the body weight; Con, control group; CS-30, 30 s chair stand test; OLST-O, one-leg standing balance test with eyes open; OLST-C, one-leg standing balance test with eyes closed; FES-I, Falls Efficacy Scale International. **, significantly greater than Pre in LRT group (*p* < 0.001); *, significantly greater than Pre in LRT group (*p* < 0.01).

## Data Availability

The data that support the findings of this study are available from the corresponding author upon reasonable request.
